# Remote continuous glucose monitoring during the COVID-19 pandemic in quarantined hospitalized patients in Denmark: A structured summary of a study protocol for a randomized controlled trial

**DOI:** 10.1186/s13063-020-04872-4

**Published:** 2020-11-25

**Authors:** Carina Kirstine Klarskov, Birgitte Lindegaard, Ulrik Pedersen-Bjergaard, Peter Lommer Kristensen

**Affiliations:** 1 Department of Endocrinology and Nephrology, Nordsjællands Hospital, Dyrehavevej 29, DK-3400 Hillerød, Denmark; 2 Department of Lung and Infectious Diseases, Nordsjællands Hospital, Dyrehavevej 29, DK-3400 Hillerød, Denmark; 3 Faculty of Health and Medical Sciences, University of Copenhagen, Blegdamsvej 3B, DK-2200 Copenhagen, Denmark

**Keywords:** COVID-19, Randomised controlled trial, protocol, CGM, Diabetes, quarantine, infection

## Abstract

**Objectives:**

Patients with diabetes are - compared to people without diabetes - at increased risk of worse outcomes from COVID-19 related pneumonia during hospitalization. We aim to investigate whether telemetric continuous glucose monitoring (CGM) in quarantined hospitalized patients with diabetes and confirmed SARS-CoV-2 infection or another contagious infection can be successfully implemented and is associated with better glycaemic control than usual blood glucose monitoring (finger prick method) and fewer patient-health care worker contacts. Furthermore, we will assess whether glucose variables are associated with the clinical outcome.

The hypothesis is that by using remote CGM to monitor glucose levels of COVID-19 infected patients and patients with other contagious infections with diabetes, we can still provide satisfactory (and maybe even better) in-hospital diabetes management despite patients being quarantined. Furthermore, the number of patient-personnel contacts can be lowered compared to standard monitoring with finger-prick glucose. This could potentially reduce the risk of transmitting contagious diseases from the patient to other people and reduces the use of PPE’s. Improved glucose control may reduce the increased risk of poor clinical outcomes associated with combined diabetes and infection.

**Trial Design:**

This is a single centre, open label, exploratory, randomised, controlled, 2-arm parallel group (1:1 ratio), controlled trial.

**Participants:**

The trial population is patients with diabetes (both type 1 diabetes, type 2 diabetes, newly discovered diabetes that is not classified yet, and all other forms of diabetes) admitted to Nordsjællands Hospital that are quarantined due to COVID-19 infection or another infection. Inclusion criteria:

1. Hospitalized with confirmed COVID-19 infection by real-time PCR or another validated method OR hospitalized with a non-COVID-19 diagnosis and quarantined at time of inclusion.

2. A documented clinically relevant history of diabetes or newly discovered during hospitalization as defined by The World Health Organizations diagnostic criteria for diabetes.

3. Written informed consent obtained before any trial related procedures are performed.

4. Male or female aged over 18 years of age.

5. Must be able to communicate with the study personnel.

6. The subject must be willing and able to comply with trial protocol.

Exclusion criteria:

1. Known hypersensitivity to the band-aid of the Dexcom G6 sensors

**Intervention and comparator:**

Participants will be randomized to either real-time CGM with the Dexcom G6, a CGM system that does not need to be calibrated, or finger-prick glucose monitoring. Blinded CGM will be mounted in the finger-prick group. In the open CGM group, the glucose values will be transmitted to a Smartdevice in the nurse office where glucose levels can be monitored remotely.

**Main Outcomes:**

The primary endpoint is the difference between groups in distribution of glucose values being in time in range (TIR), defined as 3.9 to 10 mmol/l. In addition, the primary endpoint is reported as the percentage of days of the whole admission, the patient reaches TIR. Secondary endpoints are the estimated number of saved patient-personnel contacts related to blood glucose measurements, incl. time healthcare providers spent on diabetes related tasks and PPE related tasks, during the patients’ hospitalization. Furthermore, we will assess additional glucose outcomes and associations of glucose variables and patient outcomes (As specified in the protocol).

**Randomisation:**

The service used for generating the randomization lists is www.random.org. Randomization is stratified by COVID-19 status and an allocation ratio of 1:1 to either CGM or finger-prick groups.

**Blinding (Masking):**

The design of the trial is open, however blinded CGM is recorded in the finger-prick group.

**Numbers to be randomized (sample size):**

A sample size of N=72 is required for the primary endpoint analysis based on 80% power to detect a 10% difference between groups in TIR and to allow for a 15% dropout. The 72 participants will be randomized 1:1 to open CGM or finger-prick with 36 in each group.

**Trial status:**

This structured protocol summary is based on the CGM-ISO protocol version 1.3, dated 13.05.2020. Date of first patient enrolled: 25.05.2020. Expected last recruiting is May 2021. Patients enrolled to date: 20 in total. 8 with confirmed COVID-19 infection and 12 with other infections.

**Trial registration:**

ClinicalTrials.gov Identifier: NCT04430608. Registered 12.06.2020

**Full protocol:**

The full protocol is attached as an additional file from the Trial website (Additional file 1). In the interest of expediting dissemination of this material, the familiar formatting has been eliminated; This Letter serves as a summary of the key elements of the full protocol.

**Supplementary Information:**

The online version contains supplementary material available at 10.1186/s13063-020-04872-4.

## Supplementary Information

**Additional file 1.**

## Data Availability

Trial data is stored on secure servers in the Capital Region of Denmark. The investigators will have access to the final dataset. Data will be available from the author on reasonable request at carina.kirstine.klarskov@regionh.dk

