# Supplementary material for: Remote continuous glucose monitoring during the COVID-19 pandemic in quarantined hospitalized patients in Denmark: A structured summary of a study protocol for a randomized controlled trial
Source: Trials. 2020 Nov 25;21:968. doi: 10.1186/s13063-020-04872-4 (PMC7686946; doi:10.1186/s13063-020-04872-4)
Supplement: Supplementary file 1 — Additional file 1. [file 13063_2020_4872_MOESM1_ESM.pdf]

Clinical Trial Protocol

Trial ID: CGM-ISO

Title of Trial

Remote Glucose Monitoring of Patients with Diabetes Quarantined During the COVID-19 Pandemic – a Hospital-Based Randomized Controlled Trial of the Effect of Remote Continuous Glucose Monitoring Compared to Usual Glucose Monitoring on Glycaemic Control, Diabetes Management Practice, and Patient Outcomes

Short Title: Remote Continues Glucose Monitoring During the COVID-19 Pandemic in Quarantined Hospitalized Patients

Investigational Device:	Dexcom G6
Sponsor, Funder:	Peter Lommer Kristensen
Ethical Committee number:	H-20025305
Data Protection number:	P-2020-303
Date:	13.05.2020
Version:	1.3

Confidential: This document is confidential and the property of Nordsjællands Hospital. No part of it may be transmitted, reproduced, published, or used without prior written authorization from the institution.

Statement of compliance: This document is a protocol for a clinical research study. The study will be conducted in compliance with all stipulations of this protocol, the Helsinki Declaration (2002), the EU Directive on the GCP and ICH-GCP guidelines, the Regional Scientific Ethical Committee and the Data Monitoring Board.

Sponsor and Principal Investigator: Peter Lommer Kristensen, MD, PhD, Chief Physician, Department of Endocrinology and Nephrology at Nordsjællands Hospital (NOH), Dyrehavevej 29, 3400 Hillerød, Tlf: 48296454.

Coordinating Investigator*:* Carina Kirstine Klarskov, MD, research fellow, Department of Endocrinology and Nephrology, NOH, Dyrehavevej 29, 3400 Hillerød, Tlf: 48294829.

Co-investigator: Ulrik Pedersen-Bjergaard, Professor, Chief Physician, Department of Endocrinology and Nephrology, NOH, Dyrehavevej 29, 3400 Hillerød, Tlf: 48294810.

Collaborator: Birgitte Lindegaard, Associated Professor, ph.D, Research Responsible Chief Physician, Department of Pulmonary- and Infectious Diseases, NOH, Dyrehavevej 29, 3400 Hillerød, Tlf. 48293874.

Signatures:

**Sponsor/ Investigator:**	**Name**	**Peter Lommer Kristensen**
	**Title**	**MD, Ph.D, Chief Physician**
	**Department**	**Endocrinology and Nephrology**
	**Address:**	**Dyrehavevej 29, 3400 Hillerød**
	**Telephone:**	**48296454 or 40845013**
	**E-mail:**	**Peter.lommer.kristensen.01@regionh.dk**

It is hereby declared that the trial will be performed according to the latest approved trial protocol, ICH-GCP Guidelines and local regulatory requirements and legislation

Signature: ______________________ Date: 18/5 – 2020

**Protocol Author:**	**Name**	**Carina Kirstine Klarskov**
	**Title**	**MD, clinical assistant**
	**Department**	**Endocrinology and nephrology**
	**Address:**	**Dyrehavevej 29, 3400 Hillerød**
	**Telephone:**	**48294829**
	**E-mail:**	**Carina.kirstine.klarskov@regionh.dk**

Signature: ______________________ Date: 18/5 – 2020

Contents

List of Abbreviations 5
1. Protocol Synopsis / Summary 6
2. Background / Rationale 7
   2.1 Disease background and current treatment modalities 7
   2.2 Trial rationale 8
   2.3 Benefit-risk assessments and ethical considerations 8
3. Hypothesis 8
   3.1 Primary objective 8
   3.2 Secondary objectives 9
   3.3 Primary endpoint 9
   3.4 Secondary endpoints 9
4. Trial design 9
   4.1 Summary of trial design 9
   4.2 Trial Schedule 10
   4.3 Discussion of Design 10
5. Trial population 10
   5.1 Inclusion criteria 11
   5.2 Exclusion criteria 11
6. Trial Device 11
   6.1 Investigational Device 11
7. Procedures 11
   7.1 Visit Schedule and Trial Procedures 12
   7.2 Informed Consent 13
   7.3 Subject ID number 13
   7.4 Demography 13
   7.5 Medical history and Concomitant disease 13
   7.6 Concomitant and previous medication 13
   7.7 Vital signs and Paraclinical Data 14
8. Assessment of safety 14
   8.1 Definitions – Adverse Event (AE) 14
   8.2 Definitions – Serious Adverse Event (SAE) / Serious Adverse Reaction (SAR) 14
   8.3 Assessments of AEs 15
   8.4 Reporting 15
9. Data handling / Data Management 15
   9.1 CRF 15
10. Statistical evaluation 16
   10.1 In general 16
   10.2 Justification of sample size/power calculation 16
   10.3 Effect analysis 16
   10.4 Analysis of safety parameters 16

CONFIDENTIAL

11. End of trial 16
11.1 Early termination of the trial 16
11.2 Subject Discontinuation 17
12. Administrative procedures....17
12.1 Source data and subject data protection 17
12.2 Financing....18
12.3 Insurance 18
12.4 Ethical Considerations 18
13. Publication....18
Appendix 1 19
Appendix 2 19

List of appendices

Appendix 1: Dexcom G6 user manual in Danish
Appendix 2: CGM outcome measures

List of Abbreviations

AE	Adverse Event
ADA	American Diabetes Association
BP	Blood Pressure
COVID-19	Coronavirus disease 2019
CRF	Case Report Form
CGM	Continuous Glucose Monitoring
eCRF	Electronic Case Report Form
GCP	Good Clinical Practice
ICH	International Conference of Harmonisation
MD	Medical Doctor
NOH	Nordsjællands Hospital
SAE	Serious Adverse Event
SAR	Serious Adverse Reaction
SUSAR	Suspected Unexpected Serious Adverse Reaction
VEK:	The Regional Committee on Biomedical Research Ethics

1. Protocol Synopsis / Summary

**Title of trial:**	**Remote Continues Glucose Monitoring During the COVID-19 Pandemic in Quarantined Hospitalized Patients**
**Trial ID:**	**CGM-ISO**
**Objectives:**	To investigate whether telemetric continuous blood glucose measurement in hospitalized patients with diabetes quarantined due to confirmed SARS-CoV-2 infection or another infection (requiring quarantine) can be successfully implemented and is associated with fewer patient-health care worker contacts and better glycaemic control than usual blood glucose monitoring (finger prick method). **Furthermore, to assess whether glucose variables are associated with the course of admission.**
**Trial design**	**A randomized controlled trial of isolated patients with diabetes admitted to Nordsjællands Hospital with or without COVID-19-pneumonia.A continuous glucose monitoring (CGM) based system with transmission of glucose data to a central system is used for remote monitoring of glucose levels and compared tostandard finger-prick glucose. Blinded (to patients) CGM is mounted in the finger-prick group.**
**Trial population**	**The trial population is adult diabetes patients that are isolated with or without COVID-19 infectionat Nordsjællands Hospital. Up to 100 subjects are expected to be included in a 12-month period.**
**Methods**	**A CGM system that consists of a sensor (inserted in the subcutaneous space), a transmitter connected to the sensor and a receiver, which can be a smartphone. From a "smart device" (i.e. another smartphone), glucose levels will be collected and assessed from outside the isolated patients' rooms**.
**Trial endpoints**	**The primary endpoint is the distribution of Time In Range (TIR) between groups. In addition, the primary endpoint is reported as the percentage of days of the whole admission, the patient reaches TIR.**<br><br>**Secondary, we want to estimate number of patient-health care professional contacts (associated with diabetes) and to describe glucose variations and diabetes management during admission.**<br><br>**To asses associations of glucose variables and admission course we will perform a logistic regression analysis with patient status (dead/alive) as dependent variable and glucose variables as explanatory variables and adjust for gender, age, and type of diabetes and linear regression analysis on length of hospitalization as dependent variable and glucose variables as explanatory variables and adjust for gender, age, and type of diabetes.**
**Trial device**	**Dexcom G6 sensor-based glucose monitoring system**
**Trial schedule**	**Planned first subject first visit:** April 2020<br>**Planned last subject first visit** April 2021<br>**Planned last subject last visit:** May 2021<br>**End of trial** May2021

CONFIDENTIAL

2. Background / Rationale

2.1 Disease background and current treatment modalities

Epidemics and pandemics are a constant threat to health care systems globally. This stresses the importance of preparadness for a large amount of hospitalized quarantined patients in isolation, with the extra challenges it brings. The COVID-19 pandemic challenges the Danish health care system in many aspects: An increased number of citizens are expected to be admitted to hospital due to COVID-19 infected pneumonia and this will demand extra workforce resources, extra use of protective equipment (gowns, masks, gloves, etc) and extra time used for taking protective equipment on and off. In concert these extra demands will drain the health care system and any initiative to reduce these challenges is needed.

Recent studies have showed that people with diabetes have increased risk of hospitalization due to infections (e.g. respiratory infections) compared to people without diabetes (Benfield T et al. Diabetologia, 2006). In accordance with this, new COVID-19 studies have shown that having diabetes is associated with increased risk of in-hospital death due to COVID-19 (Zhou F, Lancet, 2020, epub ahead of print). It is well known, that hyperglycaemia and hypoglycaemia in hospitalized patients, is associated with both increased mortality and increased morbidity (Silmara AO, Diabetol Metab Syndr. 2010;2:49). Therefore, the American Diabetes Association suggests that glucose levels during hospitalization do not exceed 10 mmol/l (Diabetes Care. 2019, 42: S173-S181). Obtaining such a goal can be challenging and rely on very frequent glucose measurements (at least 5 times / 24 h). This is troublesome, especially in patients that are isolated, since it demands a health care professional to enter the patient room very frequently – every time putting on and taking off protective equipment and doing cleansing procedures of hands, blood glucose meters and other equipment and still with a risk of being infected or bring contagious COVID-19 particles from one patient to a non-infected person. The prevalence of diabetes in hospitalized patients with COVID-19 has been reported as high, ranging from 10 to 33% (Wang D et al, JAMA, 2020, Zhou F, Lancet, 2020, and Arentz M et al, JAMA, 2020; all epub ahead of print).

There is an unmet need to measure and register blood glucose of isolated patients with diabetes in general, both during this pandemic, but also in the future in an easier and more secure (non-contagious) way. The use of continuous glucose monitoring (CGM) systems, with Dexcom G6, can be part of the solution, since this technology can monitor interstitial glucose levels via a sensing electrode placed in the subcutaneous space and transmit the glucose data from the patient to a surveillance unit outside the patient room. In that way the number of times a health care professional must measure glucose bed side will be dramatically reduced. This will:

1. Reduce workload for the staff (which can be directed to other patients/tasks).
2. Decrease the risk of passing on COVID-19 or other infections from a diabetes patient to other people (including staff which is key during a pandemic).
3. Reduce need and expenses for protective equipment and the considerable time used for taking the equipment on and off.
4. Provide the clinician with comprehensive data on glucose excursions during the admission, thereby enabling a better diabetes care, which may reduce the deleterious effects of the COVID-19 and other infections and reduce length of admission.

Furthermore, gathering glucose information with the CGM system will provide us with a detailed insight in glucose trajectories during hospitalization of diabetes patients something which has not systematically been reported before. It will also give us the opportunity to explore the effect of glucose variables on outcomes of COVID-19 infected diabetes patients. This has not been done before. Moreover, implementation of the CGM system will teach us important lessons about strengths and pitfalls of remote glucose monitoring in in-patients, which will be extremely valuable in the future planning of similar CGM systems in both non COVID-19 in-patients (e.g. in patients with stroke) and during future epidemics with quarantined patients.

2.2 Trial rationale

To investigate whether telemetric continuous blood glucose measurement in hospitalized patients with diabetes quarantined due to confirmed SARS-CoV-2 infection or another infection (requiring quarantine) can be successfully implemented and is associated with fewer patient-health care worker contacts and better glycaemic control than usual blood glucose monitoring (finger prick method). Fewer patient-health care worker contacts may reduce the spread of infection in the hospital, the consumption of protective equipment and time used for blood glucose measuring. With better glycaemic control during admission we may reduce length of stay at hospital, since glycaemic dysregulation during hospitalization is associated with poorer patient outcome. From Figure 1 it can be appreciated that the glucose measurements done by the Dexcom G6 sensor can be transmitted to a smartphone in the patient room (using Bluetooth) to a receiver smart phone outside the patient room (using wifi).

Figure 1

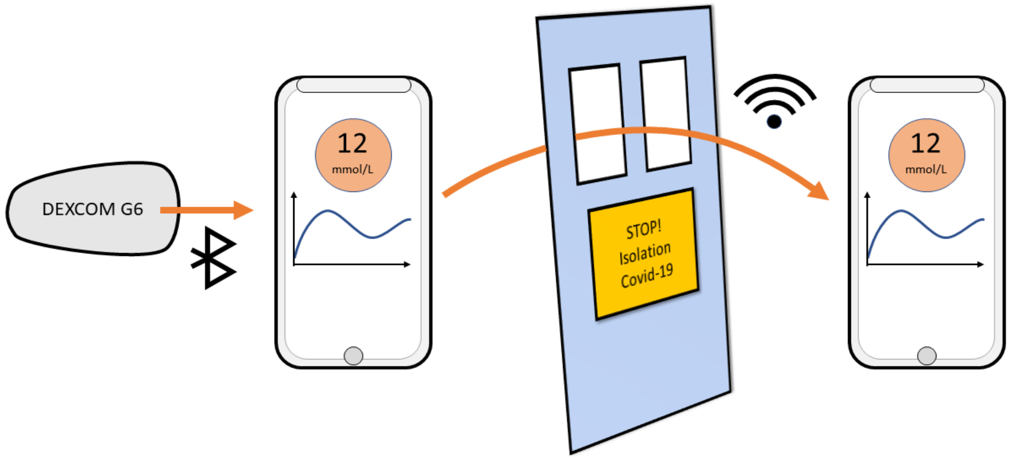

2.3 Benefit-risk assessments and ethical considerations

The risks for the patients associated with participation in the trial are considered minimal, since CGM and the trial device Dexcom G6 is currently in use for the same purposes in diabetes departments (for outpatients) around the world. The standard glycaemic target is kept unaltered during the trial, that is 5 – 10 mmol/L (which is the inhospital guideline ifrom Region Hovedstaden). Patients might get a better diabetes care during the trial since the glucose level is monitored more closely than during usual care. The general discomfort to the patients includes the application of the sensor in the skin and the discomfort of the band-aid.

3. Hypothesis

3.1 The hypothesis is that by using remote CGM to monitor glucose levels of COVID-19 infected patients with diabetes, we can still provide satisfactory (and maybe even better) in-hospital diabetes management despite patients being quarantined. Furthermore, the number of patient-personel contacts can be lowered compared to standard monitoring with fingerprick glucose collected from isolated, hospitalized, COVID 19 and non-COVID infected control groups. This reduces the risk of passing on COVID-19 from the patient to other people and reduces the use of PPE's. Improved glucose control may reduce the increased risk of poor clinical outcomes associated with combined diabetes and infection. Primary objective

The primary objective is to describe the ultimate goal of diabetes management, that is how many patients reach the glycaemic goals.

3.2 Secondary objectives

1) To estimate the number of saved patient-personnel contacts related to blood glucose measurements, incl. time healthcare providers spent on diabetes related tasks and PPE related tasks, during the patients' hospitalization.
2) To describe glucose variations during hospitalizationin our study populations.
3) To describe daily diabetes related blood glucose lowering interventions in our study populations.
4) To describe sensor performance.
5) To assess whether glucose variables are associated with the course of admission.
6) To explore differences between the different groups in regard to glucose control and outcomes like differences in glucose control and differences after transfer to an intensive care unit.

3.3 Primary endpoint

The primary endpoint is the difference between groups in distribution of time in range (TIR). In addition, the primary endpoint is reported as the percentage of days of the whole admission, the patient reaches TIR.

3.4 Secondary endpoints

1) We report the estimated number of saved patient-personnel contacts related to blood glucose measurements, incl. time healthcare providers spent on diabetes related tasks and PPE related tasks, during the patients' hospitalization. We do that by counting actual performed finger prick glucose measuremensts in the diabetes control groups (hospitalized with or without COVID-19 infection), All glucose measurements are recorded in the electronic patient records, Sundhedsplatformen.

2) Additional glucose outcomes (be based on data from Dexcom G6) are for example Time Above Range (TAR), Time Below Range (TBR), average glucose, coefficient of variation, etc. according to current consensus, see appendix 2 (T. Battelino et al., Diabetes Care, 2019 and ADA, Diabetes Care, 2019).

3) We report the glucose lowering therapy during admission (non-insulin based therapy (broken down to different drug classes), basal insulin only, basal-bolus therapy, other insulin regimens) and number of times that sliding scale insulin (including dose of insulin) has been administered for each patient. The clinical relevance and correctness (according to current guidelines in Region Hovedstaden) of the insulin administration and dose will be evaluated.

4) Sensor performance will be reported as number of technical errors per sensor during the sensors' lifetime (10 days), percentage of time with "no data" and number of premature sensor replacements due to technical sensor related errors.

5) Patient outcomes: In hospital death (yes/no), length of stay at hospital, need for respiratory support (yes/no) and intensive care (yes/no), recovered vs. fatal (death within 60 days from admission).

6) To assess associations of glucose variables and patient outcomes (in hospital death (yes/no), length of stay at hospital, need for respiratory support (yes/no) and intensive care (yes/no)) we will perform a logistic and linear regression analysis with patient status as dependent variable and glucose variables as explanatory variables and adjust for gender, age, and type of diabetes. For all measures comparisons between groups will be done using standard statistics. The following groups are predefined: in hospital death during study (yes/no), recovered vs. fatal (death within 60 days from admission), need for respiratory support (yes/no) and intensive care (yes/no)).

4. Trial design

4.1 Summary of trial design

A randomized controlled trial of patients with diabetes (both type 1 diabetes, type 2 diabetes, newly discovered diabetes that is not classified yet, and all other forms of diabetes) admitted to Nordsjællands Hospital (NOH) that are quarantined due to COVID-19 infection or another infection. We will – in a randomized sequence - monitor glucose levels in patients over 18 years of age with continuous glucose meters (CGM) or with standard fingerprick glucose. Patients are stratified by COVID-19 status (see Figure 2). It is expected that approximately 500 patients with COVID-19 will be admitted to NOH during the COVID-19 pandemic. Based on data from initial studies, 50 to 165 of these are expected to have diabetes and can therefore potentially be included in this study

and up to 100 isolated COVID-19 patients are expected to be included over a 12-month period, Other quarantined patients (non COVID-19 patients) will also be included.
Patients in this trial can be included in almost any other COVID-19 trial since the mounted sensor (1,5 cm long) will most likely not interfere with these trials Data will be collected from the electronic patient records at baseline and up to 12 months after inclusion. Data from CGM will be collected each time a patient is either discharged from the hospital or every 10th day when the sensor is exchanged. Finger prick glucose is collected from Sundhedsplatformen.

Figure 2

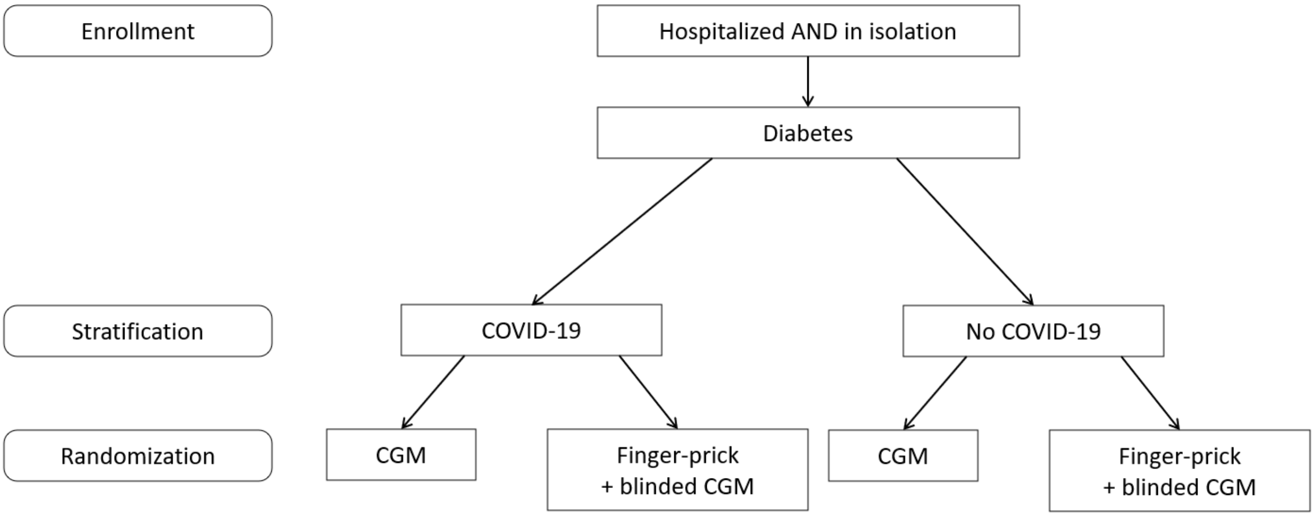

4.2 Trial Schedule

Planned first subject first visit:	April 2020
Planned last subject first visit	April 2021
Planned last subject last visit:	May 2021
End of trial	May 2021

4.3 Discussion of Design

Due to the uncertain circumstances of the development of the COVID-19 spread in Denmark the number of patients hospitalized with diabetes and COVID-19-pneumonia is unpredictable. Therefore, the design is concentrated on isolated diabetes patients both with and without COVID-19. This will provide important information on glucose management in using CGM in COVID-19 infected patients but also important data on CGM in general for patients in isolation due to any infection, and especially in preparation for the second and future waves of COVID-19 and future epidemics. Dexcom G6 was chosen as it is approved for treatment decisions regarding glucose lowering therapy.

5. Trial population

The trial population is adult isolated diabetes patients with or without COVID-19 infection that is hospitalized at NOH. Participants for the study will be identified by nurses and physicians at Nordsjællands Hospital that are in contact with eligible patients as part of their normal clinical activities. Participants can also be identified by the hospital diabetes team when they during their normal daily activities treat patients with diabetes. The emergency diabetes team at NOH is involved daily in the assessment of patients with diabetes admitted to various departments in the hospital including COVID-19 wards. Patients will be recruited from either COVID-19 wards, Lung and Infection disease wards or the Endocrinology, Nephrology and Cardiology ward. The first study contact will be by a study nurse or study doctor that are trained in the protocol and have GCP experience. The information meeting (when including participants) will be held by the same trained CGM-ISO project nurses or CGM-ISO project physician. The meeting will be held bedside in the isolation room and can if the potential participants wants, include the potential participants nurse in person and also a family member or friend by electronic media.

Subjects meeting all the inclusion criteria listed and none of the exclusion criteria will be considered eligible for the trial.

5.1 Inclusion criteria

1. Hospitalized with confirmed COVID-19 infection by real-time PCR or another validated method OR hospitalized with a non-COVID-19 diagnosis AND in isolation at time of inclusion.
2. A documented clinically relevant history of diabetes or newly discovered during hospitalization.
3. Written informed consent obtained before any trial related procedures are performed.
4. Male or female aged over 18 years of age.
5. Must be able to communicate with the study personnel.
6. The subject must be willing and able to comply with trial protocol.

5.2 Exclusion criteria

1. Known hypersensitivity to the band-aid of the Dexcom G6 sensors

6. Trial Device

6.1 Investigational Device

The investigational device is a CGM Dexcom G6. The Dexcom G6 System is intended to replace fingerstick blood glucose testing for diabetes treatment decisions. The device-system consists of a sensor, the Dexcom G6 device/sender, and connects to a smart device like a cellphone. The Dexcom G6 CGM system is probably the most precise system on the market and with no need for daily calibration with finger prick glucose. The Dexcom G6 sensor can last for 10 days without calibration and is approved for diabetes treatment decision making. Dexcom G6 has been extensively tested and is safe and approved even for pregnant women.

The CE Marking confirms that the G6 system meets the Essential Requirements of the Medical Device Directive MDD 93/42/EEC as amended by 2007/47/EC.

Please visit appendix one for a complete overview and user manual of the Dexcom G6 device.

7. Procedures

7.1 Visit Schedule and Trial Procedures

In the table below, the procedures that should be performed at each visit are described.

Visit ID	Procedures to be performed at the visit
**Visit 0/1 Screening and inclusion visit (as soon as possible after admission)**	Obtain written informed consent for the trial before any other trial procedures are performed.<br>Evaluation of inclusion and exclusion criteria.<br>Baseline data from the electronic patient records:<br>Demographic data (sex, date of birth, race) and body measurements (height and weight)<br>Standard inpatient blood tests, standard diabetes related blood tests, medication status, comorbidity, medical history, vital signs, laboratory and radiology results.<br><br>CGM sensor is applied
**Visit 2A (At discharge or on the 10th day of admission)**	Sensor collection or sensor replacement (if patient is admitted for more than 10 days).<br>Calculation of number of times finger-prick glucose has been measured (data can be found in Sundhedsplatformen).<br>Collection of data from the sensor: Sensor time, sensor failures, number of shifts and the reason for these.<br>Collection of data regarding glucose level (via. Diasend): Various glucometric targets.<br>Patient status: Dead / alive, inpatient in intensive care unit, inpatient treated with respirator, inpatient treatment.<br>Follow-up on examinations (x-ray, biochemistry, other).<br>Monitoring of diabetes, endocrinological supervision.<br>Assess AEs occurring since the last visit.<br><br>New CGM sensor is applied
**Visit 2X**	As visit 2A every 10 days until the patient is discharged. X is the next letter in the alphabet.
**7 days after End of trial**	Assess AEs occurring since the last visit

7.2 Informed Consent

The written informed consent must be obtained (i.e. signed and dated by the subject) before any trial activities are performed.

Each subject must be informed that participation in the trial is voluntary and that he/she may withdraw from the trial at any time during the course of the trial and that withdrawal of consent will not affect his/her subsequent medical treatment or relationship with the treating physician.

Subjects meeting the inclusion criteria for the trial will be give verbal and written information about the trial. The subject will be given time to ask questions and allowed at least 24 hours to consider the trial before deciding whether to participate.

If subjects desire, they may make a later appointment with the site investigator after having reviewed the written information about the trial, at which they can meet and discuss participation in the trial. Such a meeting must take place in an undisturbed room. Under normal circumstances the subject may bring a family member or friend to aid the decision-making, however due to the current situation with COVID-19 and restrictions in visits, this will not be possible, and the subject will be informed about this. Instead it will be secured that the potential participants can connect by a phonecall or electronically with a family member or friend during the meeting from smartdevices that have electronic medias for meetings installed. The device or phone will be provided by the personnel.

The subjects will be informed of any potential risks associated with the trial.

It is the responsibility of the principal investigator or sub-investigator or personnel related to the trial (e.g. study nurses) to obtain the written informed consent from the subject.

After completion of trial, all participating subjects will be informed of the overall results. Information of individual data the interpretation hereof will be provided by the investigator. The individual subject's right not to know of own data will be respected.

By giving informed consent the participant also agrees that the investigators, the sponsor and sponsor's representatives as well as any supervisory authority can have direct access to information in the patient's record etc. including the electronic patient record, in order to see information about the subject's health conditions which are necessary as part of the implementation of the research project as well as for control and monitoring purposes, including self-monitoring, quality control and monitoring, that are required to be performed.

7.3 Subject ID number

All subjects enrolled must be identifiable throughout the trial. This will be done by using a 3-digit subject number starting at 101 and allocated to the subject at Visit 0/1 (the screening visit).

7.4 Demography

The following data will be recorded:

- Date of birth
- Ethnic origin/Race
- Gender

7.5 Medical history and Concomitant disease

Information from subjects' medical records regarding co-morbidities and intercurrent disease will be recorded at baseline and throughout the course of the trial (up to 12 months). The information is gathered from the active diagnosis list in Sundhedsplatformen and includes a diagnosis of: any lung-related disease including COPD, heart disease, hypertension, cancer, rheumatological disease, immune diseases including HIV and haematological disease.

7.6 Concomitant and previous medication

Concomitant medication is any medication, which is taken after signing the Informed Consent. Relevant previous medication will be recorded at visit 0/1. Information about concomitant medication and changes will be

collected. This includes antibiotics and antiviral medications, antidiabetic medications, immune supporting medications, inhalation medications, and circulatory supportive medications.

7.7 Vital signs and Paraclinical Data

Since vital and physical examination data are collected from the electronic patients records, we don't control under which circumstances they are performed.

The information collected is:

Height and weight
Blood pressure
Pulse
Laboratory blood test results: Haemoglobin, HbA1c, plasma glucose, C-peptide, leukocytes with differential count, CRP, platelet count, iron, transferrin, MCV, reticulocytes, albumin, ALAT, ASAT, alkaline phosphatase, LDH, bilirubin, calcium-ion, lipids, creatinine, potassium, sodium, urea. These measurements are standard measurements on hospitalized patients with diabetes. No biobank is established.
Paraclinical descriptions: X-rays/MR or CT scans descriptions to asses the severity of COVID-19 pneumonia.

Laboratory tests will vary between patients depending of the severity of their disease, however standard tests are the same for all patients admitted to Nordsjællands Hospital. The CGM-ISO team is not involved in decisions regarding this.

8. Assessment of safety

Information about Adverse Events (AEs), whether reported by the subject, discovered by the investigator by reviewing diary records, detected through physical examination, laboratory test or other means, must be collected and recorded on the AE form and followed up as appropriate.

Evaluation of AEs including severity, causality, outcome and seriousness assessments must be performed by a physician.

Any AE occurring from the time the informed consent was signed until discharge must be recorded and reported on an AE page in the CRF.

Standardised report forms for AEs and SAEs will be provided as part of the CRF.

8.1 Definitions – Adverse Event (AE)

An AE is any untoward medical occurrence in a subject administered a medicinal product/device and which does not necessarily have a causal relationship with this treatment.

The following events should be recorded as AEs:

- Diabetes related AE's will be recorded.

The following events should not be recorded as AEs:

- All other adverse events.

8.2 Definitions – Serious Adverse Event (SAE) / Serious Adverse Reaction (SAR)

A serious adverse event/reaction is an event that a:

- Results in death
- Is life-threatening - this refers to an event in which the subject was at risk of death at the time of the event
- Requires in-subject hospitalisation or prolongation of existing hospitalisation
- Results in persistent or significant disability or incapacity
- Is a congenital anomaly or birth defect
- Is judged medical important (this refers to an event that may not be immediately life-threatening or result in death or hospitalisation, but may jeopardise the subject or may require intervention to prevent one of the other outcomes listed above)

- In relation to trial safety, no SAE or SUSAR will be recorded.

8.3 Assessments of AEs

Severity:

The severity of an AE is a clinical observation assessed by the investigator using the following definitions:

- Mild: Transient symptoms, no interference with the subject's daily activities
- Moderate: Marked symptoms, moderate interference with the subject's daily activities
- Severe: Considerable interference with the subject's daily activities, unacceptable

Causality / Causal Relationship to IMP:

The following terms and definitions are used when assessing the relationship between an AE and the relevant trial product (IMP):

- Probable: good reason for sufficient documentation to assume a causal relationship
- Possible: a causal relationship is conceivable and cannot be dismissed
- Unlikely: the event is most likely related to aetiology other than the trial product

Final outcome:

The outcome of an AE is assessed by the Investigator using the following definitions:

- Recovered/resolved: Fully recovered or has returned to baseline
- Recovered with sequelae: As a result of the AE the subject suffered persistent and significant disability/incapacity
- Not recovered: The condition has not returned to baseline, however symptoms may have improved
- Fatal: Event that results in death.
- Unknown: The outcome is unknown. This term should only be used when no other definition is possible e.g. the subject is lost to follow-up

8.4 Reporting

AE reporting will be performed in the CRF – and reviewed by:

Contact person: Peter Lommer Kristensen

Email address: Peter.lommer.kristensen.01@regionh.dk

Emergency phone: +45 40845013 (Reachable 24/7)

9. Data handling / Data Management

Data will be stored for 5 years, after which they will be stored in an anonymised format.

The Data Protection Regulation and the Data Protection Act is complied with.

9.1 CRF

A CRF is provided and all data related to the trial will be recorded in here.

The CRF is to be completed by the investigator at the time of the subject's visit to the clinic so that it always reflects the latest observations for the subject.

At the subject's final visit, the CRF should be verified and signed off by the responsible investigator at the site.

10. Statistical evaluation

10.1 In general

Statistical analysis will be conducted by the Investigators (responsible: Carina Kirstine Klarskov, in close collaboration with a statistician).

All data will be described including data-incompleteness as well as reasons for data-incompleteness. Data will be analysed by Carina Kirstine Klarskov and Peter Lommer Kristensen. Any changes to the statistical analysis plan will be described in any future publications.

The primary analysis will be based on all subjects included in the study and the primary endpoint analysis will be based on intention-to-treat with all subjects analyzed in the group to which they were randomized. The primary endpoint is difference in glyceamic control based on CGM readings of time in range (TIR).
Preplanned subgroup analyses will be performed separately for COVID-19-infection (yes/no). Standard statistics will be used to compare primary and secondary outcomes in the two groups. Furthermore, a mixed model with analysis of covariance (ANCOVA) will be performed for primary and secondary endpoints to be able to include the possible effect of confounders on the endpoints. Relevant covariates to include in the analysis will be (but are not limited to) baseline Hba1c, COVID-19-infection (yes/no), glucose lowering treatment (insulin yes/ no).

All secondary outcome results will be adjusted for multiplicity.

10.2 Justification of sample size/power calculation

Based on previous CGM-based trials a 5% difference in TIR for outpatients is considered clinically relevant due to significant changes in complications and outcomes for every 5% change. For in-patients however this number has not been reported before. We assume that a 10% difference between groups in TIR with a SD of 14 percentage points (SD is based on combined CGM data from previous CGM-based studies in our research group on outpatients and an SD of 14 has also been reported in other CGM-based studies). This will require a sample size of 62 to achieve a power of 80% and an alpha value of 0.05. To allow for a 15% dropout 72 is planned to be randomized in this trial. Data will be reported with a 95% confidence interval, and a p-value of 0.05 or below will be considered statistically significant.

10.3 Effect analysis

Please visit primary and secondary endpoints section 3.3-3.4.

10.4 Analysis of safety parameters

A quantitative description of serious and non-serious adverse events will be presented.

11. End of trial

Within 90 days after the trial completion the Sponsor must inform the Ethics Committee about the completion. The result of the trial must be submitted within 12 months.

11.1 Early termination of the trial

The Sponsor reserves the right to terminate the trial under the following conditions:

- Safety concerns
- Proven lack of efficacy

If the trial is prematurely terminated or suspended the investigator and/or Sponsor should promptly inform the pertinent ethics committee.

11.2 Subject Discontinuation

The subject will be advised in the informed consent form that he/she has the right to withdraw from the trial at any time without prejudice. Where discontinuation from the trial is initiated by the subject, the investigator is to ascertain the primary reason for discontinuation from the list below:

- An AE for which the investigator did not consider discontinuation from the trial necessary
- Co-existing disease
- Withdrawal of consent
- Other reasons

The subject may at any time be discontinued from the trial at the discretion of the investigator

Subjects <u>must</u> be discontinued from the trial under the following circumstances:

- If a criterion equivalent to an exclusion criterion occurs
- If, in the investigator's opinion, continuation in the trial would be detrimental to the subject's well-being
- Occurrence of intolerable AE(s) as determined by the investigator and/or subject
- If informed consent is withdrawn

If a patient is moved to the intensive care unit or is no longer isolated, the sensor is not removed, but glucose measurements are no longer based on CGM-values but from standard care finger-prick. Assessment of the primary endpoint is no longer done, however data for secondary and exploratory endpoints are still collected.

In all cases, the primary reason for discontinuation must be recorded in the CRF and in the subject's medical records. Follow-up on the subject is necessary to establish whether the reason was an AE. If so, this must be reported in accordance with the appropriate procedures.
Data obtained until discontinuation will be entered in the clinical database and used for statistical analyses.

12. Administrative procedures

12.1 Source data and subject data protection

Source data will be registered in patient records or on source data sheets or directly in the CRF.

Prior to start of recording of data from subjects, the investigator will prepare a Source Data Location Agreement to document where the first recording of data is done.

As a minimum requirement, the following data must be source data-verifiable in source documentation other than the CRF:

- Subject's date of birth.
- Confirmation of participation in the trial.
- Date of informed consent.
- Confirmation of subject eligibility (in/exclusion criteria).
- Any AEs and SAEs should be described in detail.
- Date and number of each trial visit and telephone contact.

A common CRF will be constructed and provide the basis for a central database. Data will in the central database be stored in coded form according to the rules of the Danish Data Protection Agency (Videnscenter for Dataanmeldelser) with whom the trial is registered. The patient will have a unique identification number.

In accordance with the Danish Data Protection Agency data processing will be completed by 25.03.2025. Afterwards data in paper form will be destroyed and electronic data will be transferred and stored at the Danish Data Archives (Statens Arkiver).
Carina Kirstine Klarskov will be responsible for data collection and processing.
In addition, Peter Lommer Kristensen will be involved in data processing.

12.2 Financing

Financial support for the conduct of this study will be given to Sponsor's research account at Nordsjællands Hospital under the Regions CVR. No. 29190623 from Novo Nordisk Foundation who supports the trial with 1.105.755kr and Grosserer L.F. Foughts Foundation with 129.500kr.
The Primary-Investigator is not associated with any private companies or funds that have an economic interest in the research project.

12.3 Insurance

Patients will be covered according to current regulations by the product-responsibility-insurance for the trial medication and the law on patient-insurance.

12.4 Ethical Considerations

Irrespective of the outcome of the trial it will provide useful information on the risk of glucose excursions and the consequences hereof during a viral infection.
The result of the trial will thereby provide physicians and patients with new relevant information guiding their choice of monitoring system during future viral outbreaks.

The risks for the patients associated with participation in the trial are considered minimal, since CGM and the trial device Dexcom G6 is currently in use for the same purposes in diabetes departments around the world. The standard glycaemic target is kept unaltered during the trial. Patients might get a better diabetes care during the trial since the glucose level is monitored more closely than usual care. The general discomfort to the patients includes the application of the sensor in the skin and the discomfort of the band-aid.

13. Publication

The trial is investigator-initiated and data are owned by Region Hovedstaden. Both positive, negative and in-conclusive study results will be published in international peer-reviewed scientific journal by the investigators of the study group and at www.clinicaltrials.gov. Carina Kirstine Klarskov will draft the first manuscript.
The Vancouver guidelines will be applied.

Investigator Signature

It is hereby confirmed that we at our department will be participating in this project according to the protocol and that we by the sponsor will be funded according to the budget

Investigator	______________________	Date	18/5 – 2020
	Signature		DD-MMM-YYYY

Appendix 1

Appendix 2

CONFIDENTIAL

Appendix 1

Dexcom G6®

START HER

Opsæt

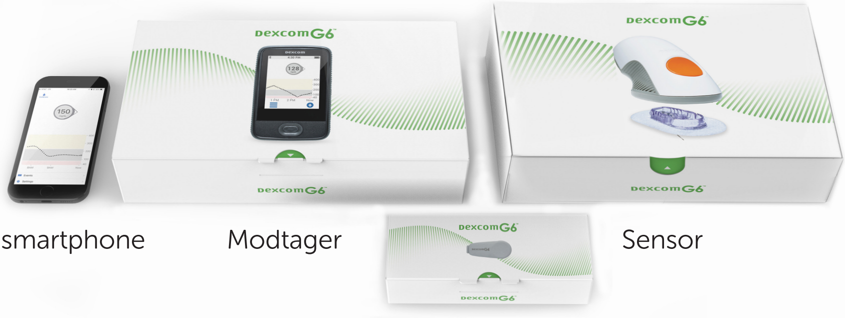

Din smartphone

Modtager

Sensor

Sender

Brugervejledning

G6 Oversigt

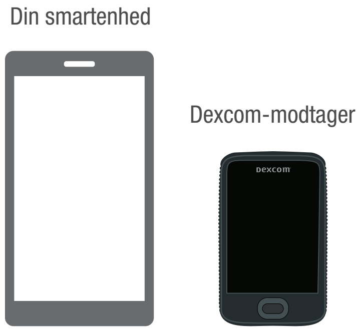

Skærmenhed

- Viser glukoseoplysninger
- Opsæt din smartenhed, Dexcom-modtager (valgfrit i visse områder) eller begge
- Du kan se en liste over smartenheder og operativsystemer, der i øjeblikket er kompatible, på: dexcom.com/compatibility

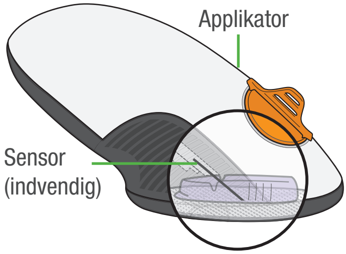

Applikator med indbygget sensor

- Sensoren henter glukoseoplysninger
- Sensorapplikatoren indfører sensoren under din hud

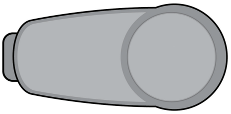

Sender

- Sender glukoseoplysninger fra sensoren til skærmenheden

Alle tegninger er illustrative. Dit produkt kan se anderledes ud.

Gennemgå sikkerhedserklæringen *Sådan bruger du din G6, kapitel 2,* før du bruger din G6.

Enhedens funktion

G6 sender glukoseoplysninger (G6-aflæsninger) til din skærmenhed

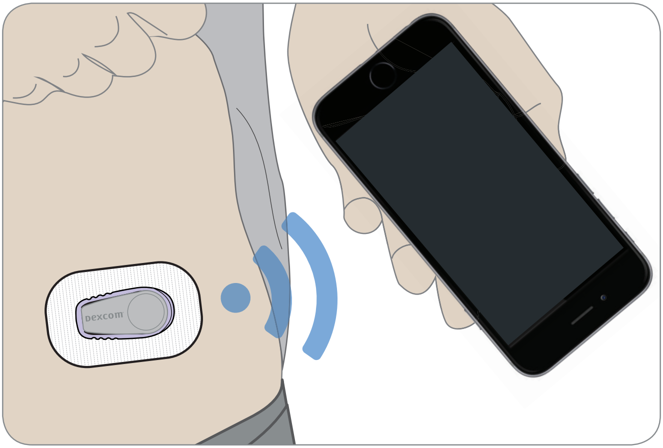

Brug fanerne herunder til at opsætte appen eller modtageren

Vil du opsætte begge? Vælg én, der skal opsættes først, og gå til den pågældende fane. Det sidste trin viser, hvordan du opsætter den anden skærmenhed. Brug ikke begge faner.

Andre måder, hvorpå du kan lære, hvordan du opsætter din G6:

- Se vejledningen online på: dexcom.com/IFU/g6/international
- Kontakt din lokale Dexcom-repræsentant for at få hjælp

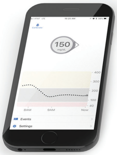

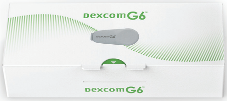

Sender

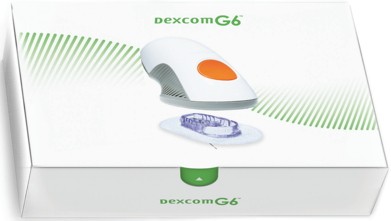

Din smartphone

Sensor

Opsæt appen

Trin 1: Opsæt appen

A Download og åbn Dexcom G6-appen

B Følg opsætningsvejledningen på skærmen

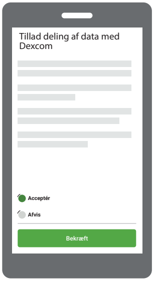

1 Når du bliver bedt om det:

- Send dine data til skyen.
  Dette giver dig mulighed for at bruge:
  - Del: Send dine G6-data til følgere.
  - KLARHED: Tal med læger om dataene; se mønstre (muligvis ikke tilgængeligt i alle områder).
- Indtast sensorkoden
  (fra den sensorapplikator, du vil indføre).
  - Ingen sensorkode?
    Se *Sådan bruger du din G6, Bilag A Fejlfinding.*

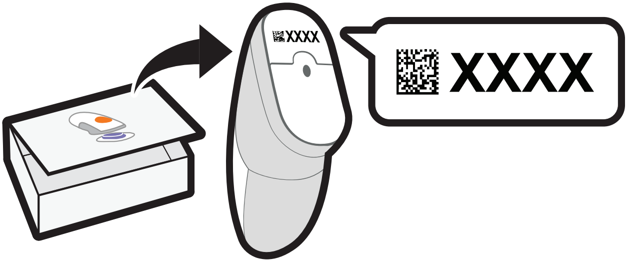

- Indtast dit serienummer (SN) fra:

Senderens æske

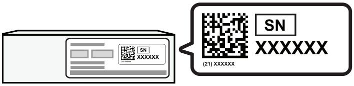

eller

Bagsiden af senderen

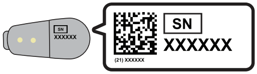

2 Kan du se den blå timer for sensoropvarmning? Det betyder, at sensoren er ved at vænne sig til din krop.

Under opvarmning:

- Ingen G6-aflæsninger, alarmer/adviseringer
- Hold smartenheden inden for en afstand af 6 meter fra senderen

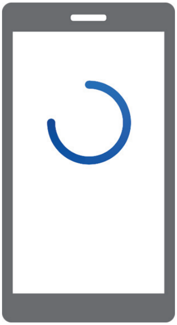

C Vent 2 timer

- Når sensoren er varmet op, skal du trykke på **OK** for at få vist startsiden
- Nu kan du modtage G6-aflæsninger, advarsler/adviseringer

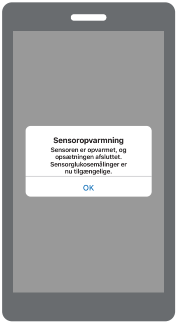

Trin 2: *Se Sådan bruger du din G6*

Lær, hvordan du:

- Læser startsiden
- Bruger alarmer og adviseringer
- Træffer beslutninger vedrørende behandling
- Foretager fejlfinding af problemer

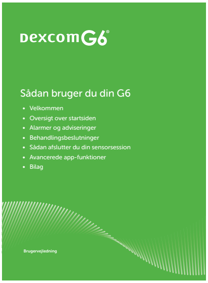

Trin 3: Valgfrit – Opsæt modtager

Tænd for modtageren ved at trykke på tænd/sluk-knappen og holde den nede i 2-3 sekunder. Følg derefter instruktionerne på skærmen.

Brug ikke fanen opsæt modtager i disse vejledninger. Trinnene på denne fane er til opsætning af modtageren før opsætning af appen.

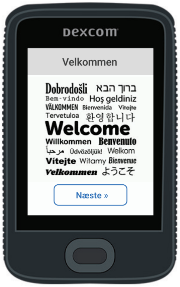

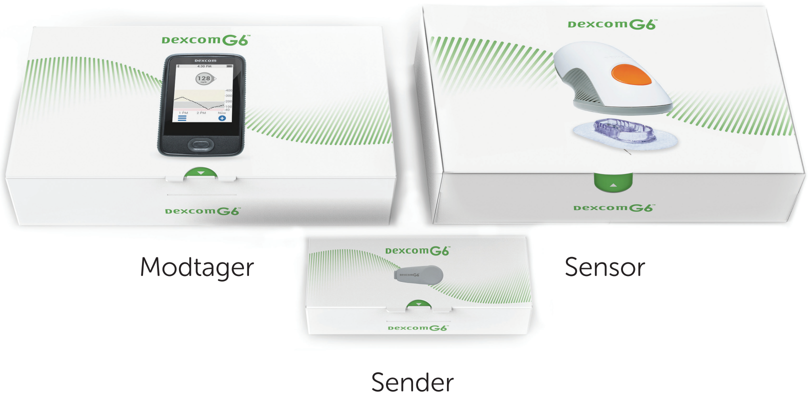

Opsæt modtager

Trin 1: Opsæt modtager

A Tag modtageren ud af æsken

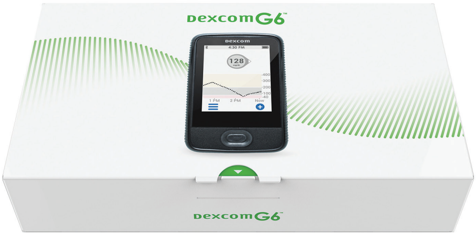

B Tænd for modtageren

Tryk på tænd/sluk-knappen, og hold den nede i 2-3 sekunder.

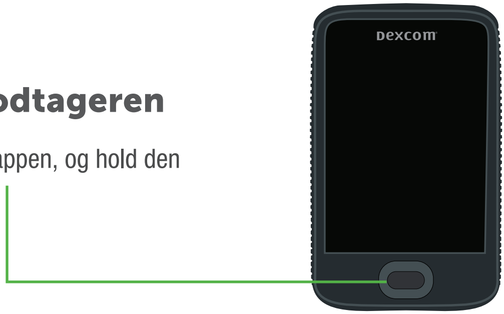

C Følg instruktionerne på skærmen

1 Når du bliver bedt om det, skal du indtaste din:

- Sensorkode (fra den sensorapplikator, du vil indføre)
  - Ingen sensorkode?
    Se *Sådan bruger du din G6, Bilag A Fejlfinding.*

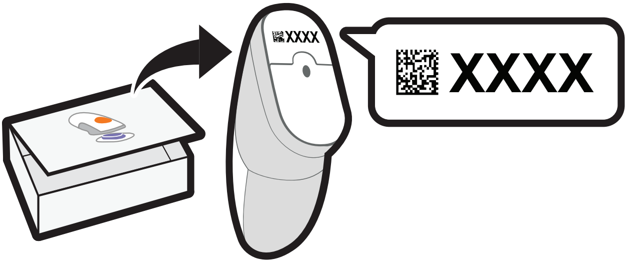

- Serienummer (SN) fra:

Senderens æske

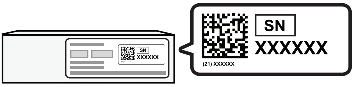

eller

Bagsiden af senderen

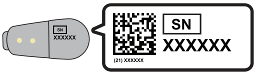

2 Du har fuldført trinnet, når du får vist følgende skærmbillede:

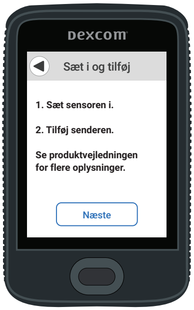

Trin 2: Brug applikatoren til at indføre indbygget sensor

A Tag applikatoren med indbygget sensor ud af æsken

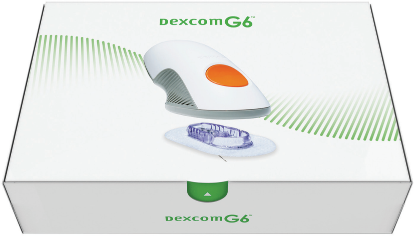

B Vælg sensorsted

18 år og derover	2-17 år

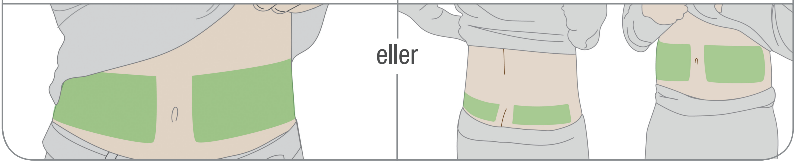

C Brug applikatoren til at indføre indbygget sensor

1 Vask hænderne, og tør dem; rengør sensorstedet med en alkoholserviet

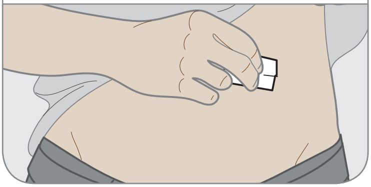

2 Brug applikatoren med sensorkoden angivet under opsætning af skærmenhed

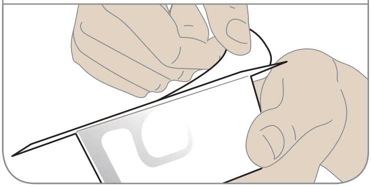

3 Fjern den klæbende bagbeklædning. Rør ikke ved klæbemidlet

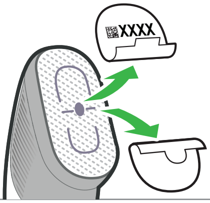

4 Flyt applikatoren til sensorstedet

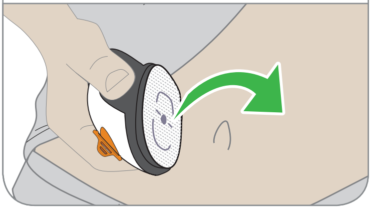

5 Placér klæbemidlet på huden

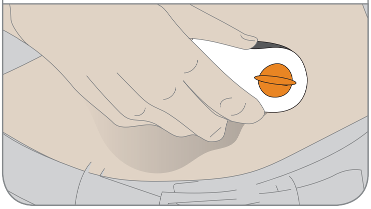

6 Fold sikkerhedsafskærmningen, og bræk den af

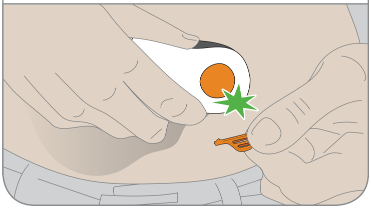

7 Tryk på knappen for at indføre

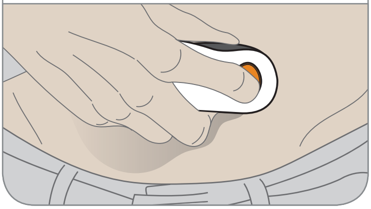

8 Kassér applikatoren (følg lokale retningslinjer)

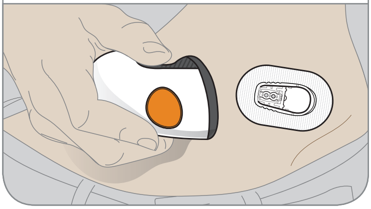

Trin 3: Fastgør sender

A Tag senderen ud af æsken

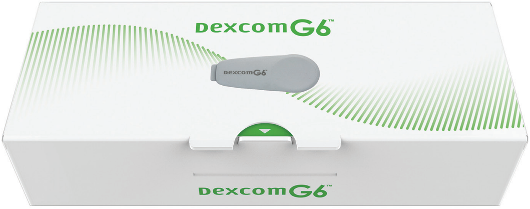

B Tryk senderen ind

1 Rengør senderen med alkohol

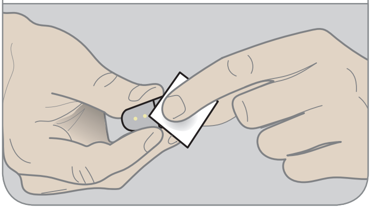

2 Sæt senderens strop ind i sprækken

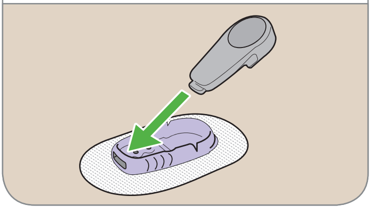

3 Tryk senderen helt på plads (der skal lyde et klik)

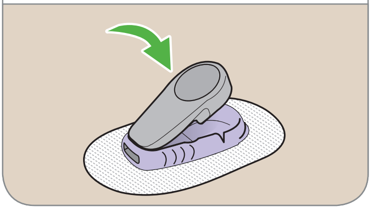

4 Gnid 3 gange rundt på plasteret

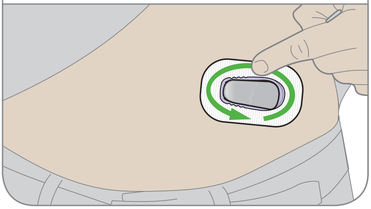

Trin 4: Start sensoren på modtageren

A Kobling mellem de 2 kan tage op til 30 minutter

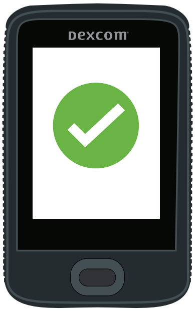

B Tryk på Start sensor for at påbegynde opvarmningen, der tager 2 timer

Under opvarmning:

- Ingen G6-aflæsninger, alarmer/adviseringer
- Hold modtageren inden for en afstand af 6 meter fra senderen

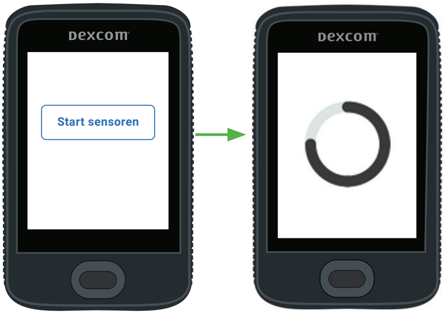

C Vent 2 timer

- Tryk på Næste for at gå til startsiden, når opvarmningen er afsluttet
- Nu kan du modtage G6-aflæsninger, advarsler/adviseringer

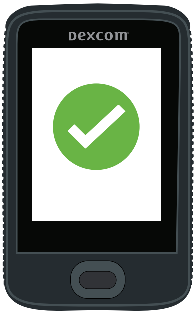

Trin 5: Se *Sådan bruger du din G6*

Lær, hvordan du:

- Læser startsiden
- Bruger alarmer og adviseringer
- Træffer beslutninger vedrørende behandling
- Foretager fejlfinding af problemer

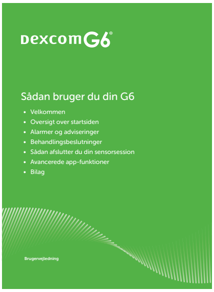

Trin 6: Valgfrit – Opsæt app

Download appen på din smartenhed, og åbn den. Følg derefter instruktionerne på skærmen.

Brug ikke fanen opsæt app i disse vejledninger. Trinnene på denne fane er til opsætning af appen før opsætning af modtageren.

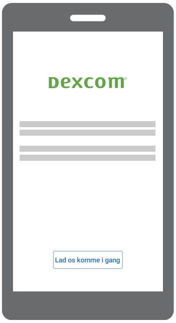

© 2018 Dexcom, Inc. Alle rettigheder forbeholdes.

Dækket af patenter dexcom.com/patents.

Dexcom, Dexcom G4 PLATINUM, Dexcom G5 Mobile CGM System, Dexcom G6, Dexcom Share, Dexcom Care er enten registrerede varemærker eller varemærker tihørende Dexcom, Inc. i USA og/eller andre lande. *Bluetooth* er et registreret varemærke, der ejes af *Bluetooth* SIG. Apple er et registreret varemærke, der ejes af Apple Inc. Android er et registreret varemærke, der ejes af Alphabet Inc. Alle andre produkt- eller virksomhedsnavne, der er nævnt i denne publikation, er handelsnavne, varemærker eller registrerede varemærker tilhørende deres respektive ejere.

Dexcom®

Dexcom, Inc.
6340 Sequence Drive
San Diego, CA 92121 USA

+1.858.200.0200
dexcom.com

Uden for USA: Kontakt din lokale Dexcom-repræsentant

EC REP

MDSS GmbH
Schiffgraben 41
30175 Hannover, Tyskland

CE
0086

LBL016323 Rev 001 MT25297

APPENDIX 2: CGM-related outcome measures (ADA, Diabetes Care, 2019, and T. Battelino et al., Diabetes Care, 2019)

TIR metrics:
Time spent In normal glucose Range (TIR).
Time glucose is Above Range (TAR).
Time glucose is Below Range (TBR).

Glucose exposure metrics:
AUC for blood glucose during periods when blood glucose levels reach hyperglycemia level 1 2 and 3.
AUC for blood glucose during periods when blood glucose levels reach hypoglycemia level 1 and 2.
Mean daytime blood glucose levels.
Mean nocturnal blood glucose levels.
eHBA1c.

Glycemic variability metrics:
SD of 24-hour blood glucose values.
SDof daytime blood glucose values.
SD of nocturnal blood glucose values.
SD MAGE (mean amplitude of glycemic excursions).
glucose variability measured by the coefficient of variation (CV).

Other metrics:
Number of hypoglycemic events in total and divided into levels and nighttime/daytime.
Number of hyperglycemic events in total and divided into levels and nighttime/daytime.

Definitions for the above section:
Levels:

<3mmol/L hypoglycemia level 2
3-3.9 mmol/L hypoglycemia level 1
3.9-10 mmol/L normoglycemia
10-13.9mmol/L hyperglycemia level 1
13.9-22.2 mmol/L hyperglycemia level 2
>22.2mmol/L hyperglycemia level 3

Furthermore, hypoglycemia level 3 is defined as a severe event characterized by altered mental state and/or physical status requiring assistance (ADA, Diabetes Care, 2019).

Hypo- and hyperglycemic events are defined as at least 15 min spent in that certain level, and each event must be at least 30 min apart.
Nighttime is defined as midnight – 6.am. **Daytime** is defined as 6.am – midnight.
